# Supplementary material for: Rescue of morphological defects in Streptomyces venezuelae by the alkaline volatile compound trimethylamine
Source: Microbiol Spectr. 2024 Aug 21;12(10):e01195-24. doi: 10.1128/spectrum.01195-24 (PMC11448094; doi:10.1128/spectrum.01195-24)
Supplement: Supplemental material — Fig. S1 to Fig. S21. [file spectrum.01195-24-s0001.pdf]

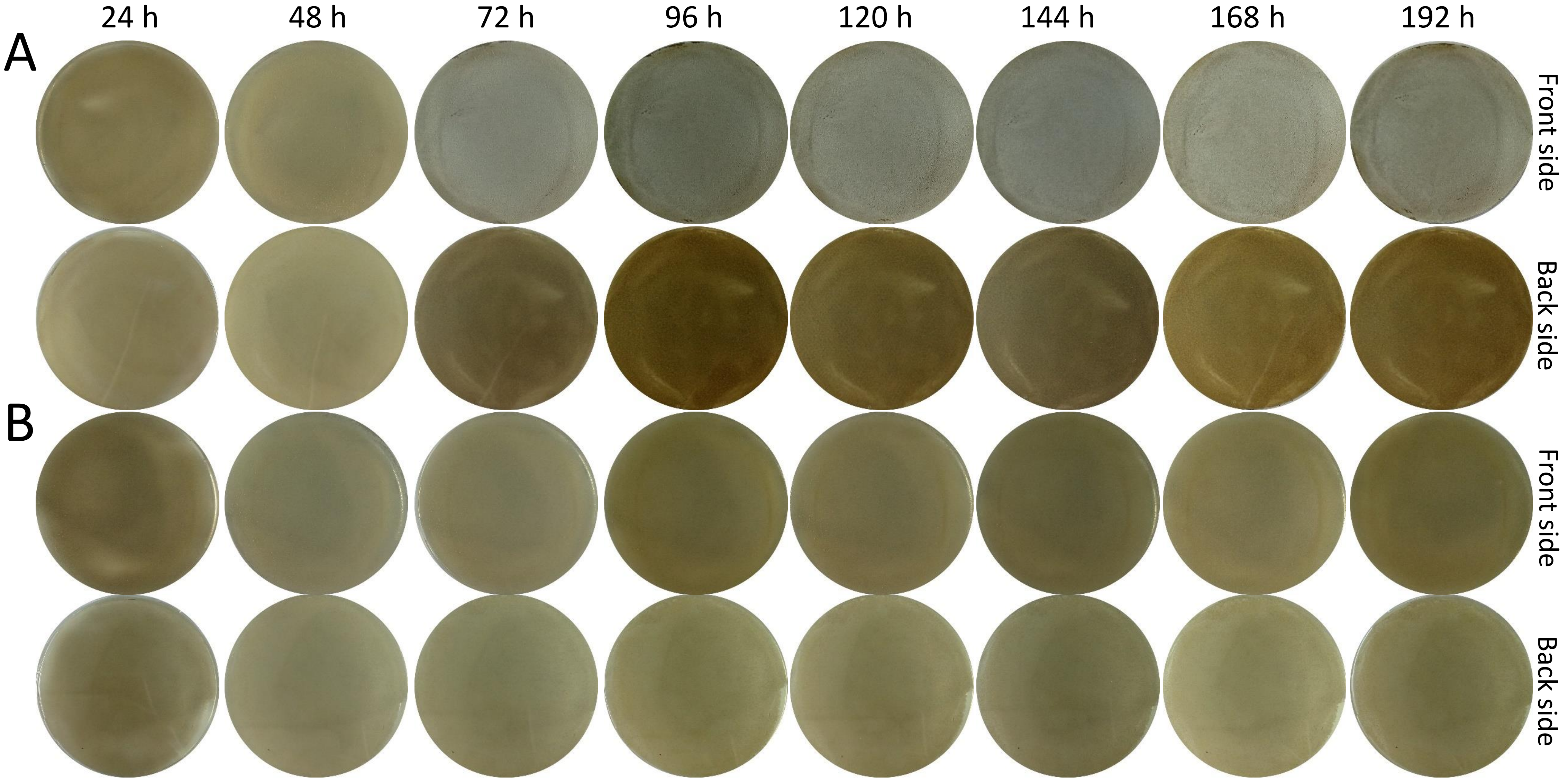

Fig. S1. Phenotypes of the *S. venezuelae* wild-type strain ISP5230 (A) and the mutant strain MU-1 (B). ISP5320 (WT) and MU-1 were grown on solid N-Evans-CA in separate plates for the indicated times before photographing.

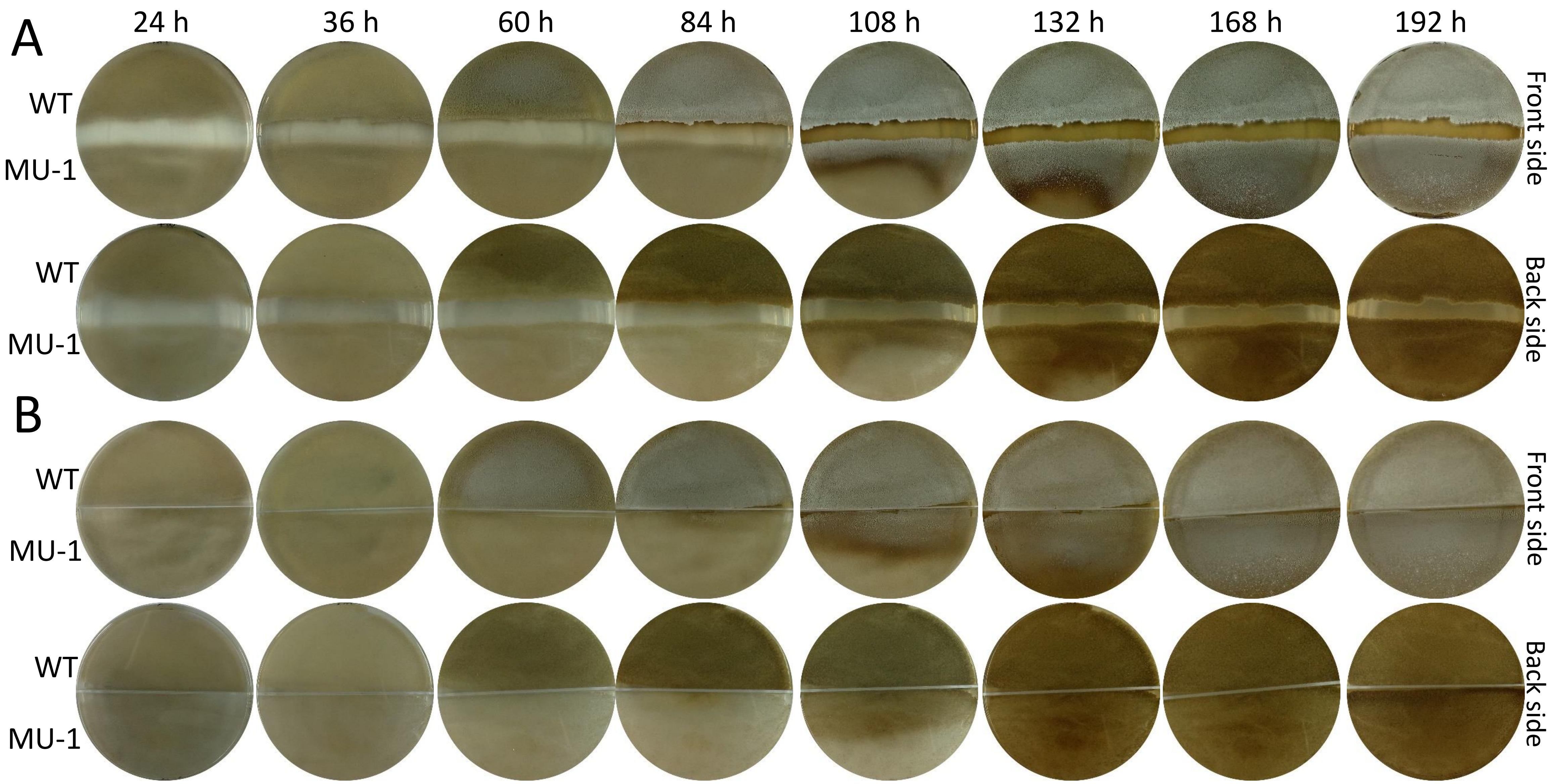

Fig. S2. Co-incubation analysis of the *S. venezuelae* wild-type strain ISP5230 and the mutant strain MU-1 on petri dishes without (A) and with (B) physical separation. ISP5320 (WT) and MU-1 were grown on solid N-Evans-CA for the indicated times before photographing.

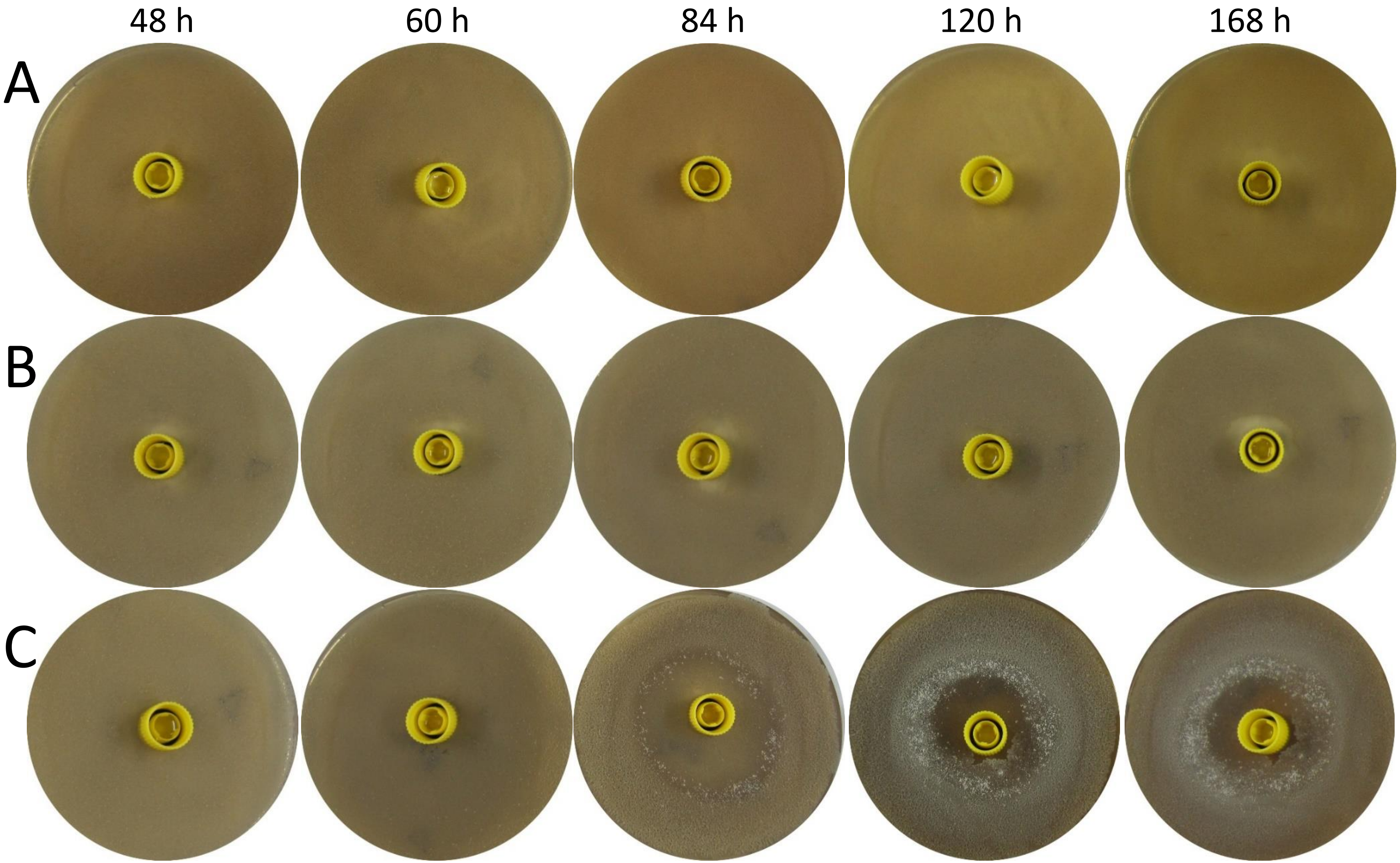

Fig. S3. Impacts of exposure to ammonium (A), water (B), and TMA (C) on the growth of strain MU-1 grown at 30°C on solid N-Evans-CA medium. Images were taken at the indicated growth times.

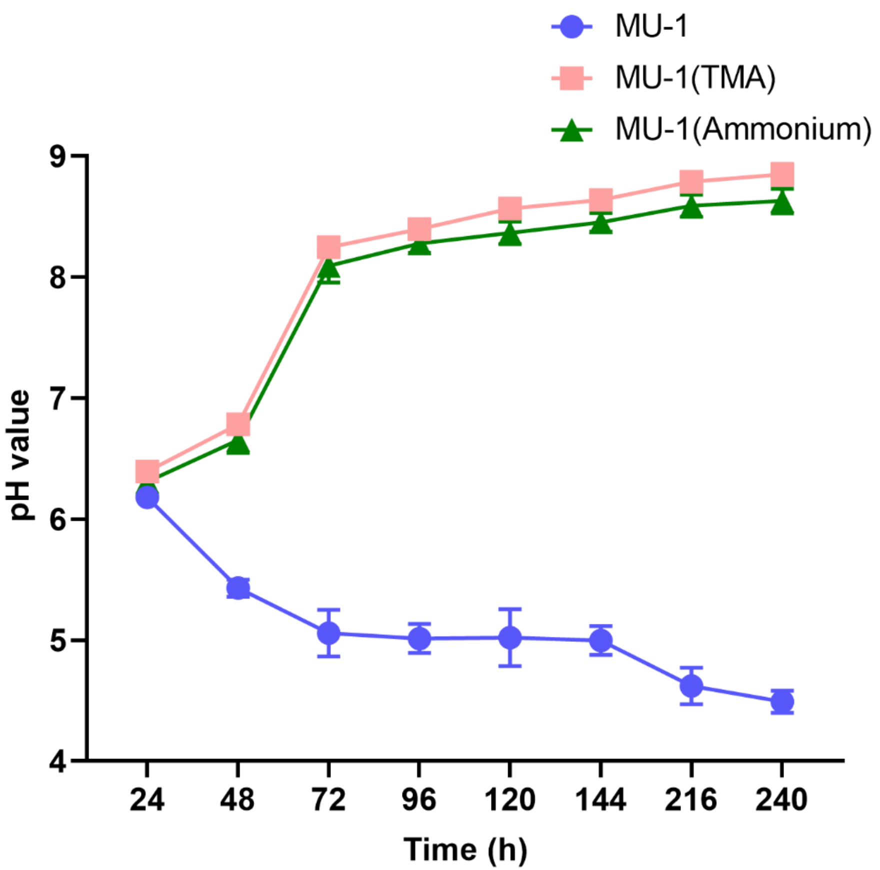

Fig. S4. The pH change over time after exposure to water, ammonium, and TMA for the growth medium of strain MU-1 grown at 30° C on solid N-Evans-CA medium.

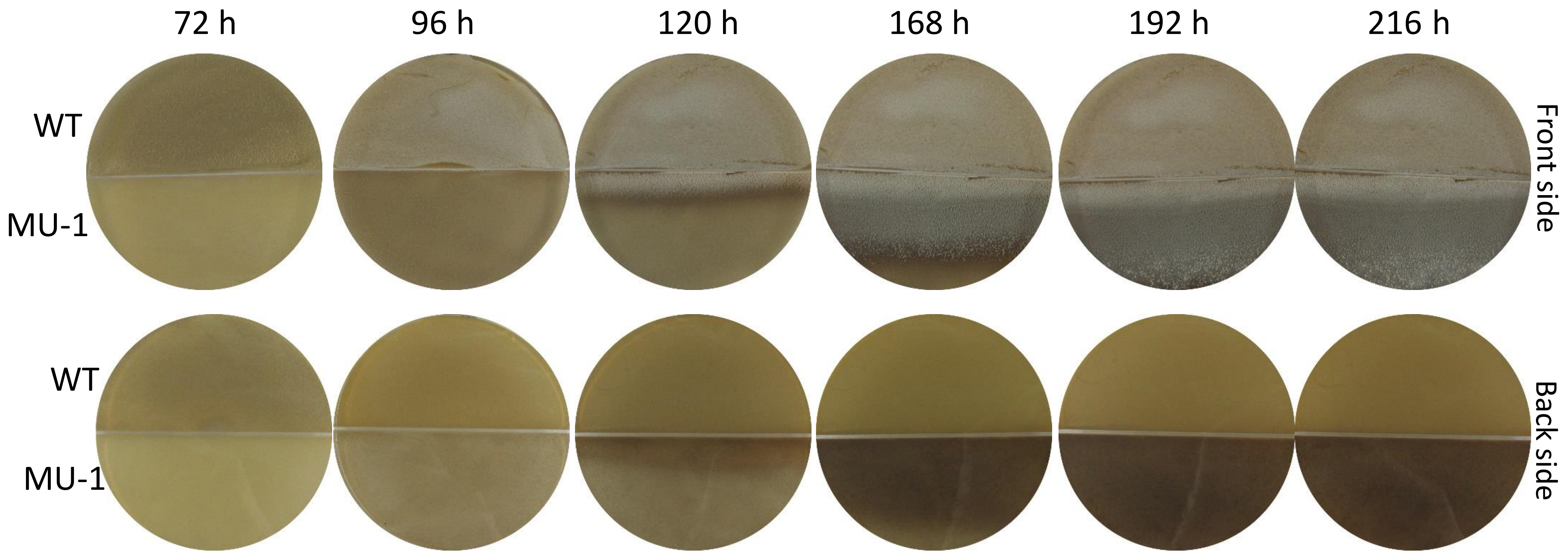

Fig. S5. Impact of the growth medium on the ability of the *S. venezuelae* wild-type strain ISP5230 to rescue MU-1**.** ISP5230 (WT) was grown on solid YBP and MU-1 was grown on solid N-Evans-CA medium in a petri dish with a physical barrier for the indicated times before photographing.

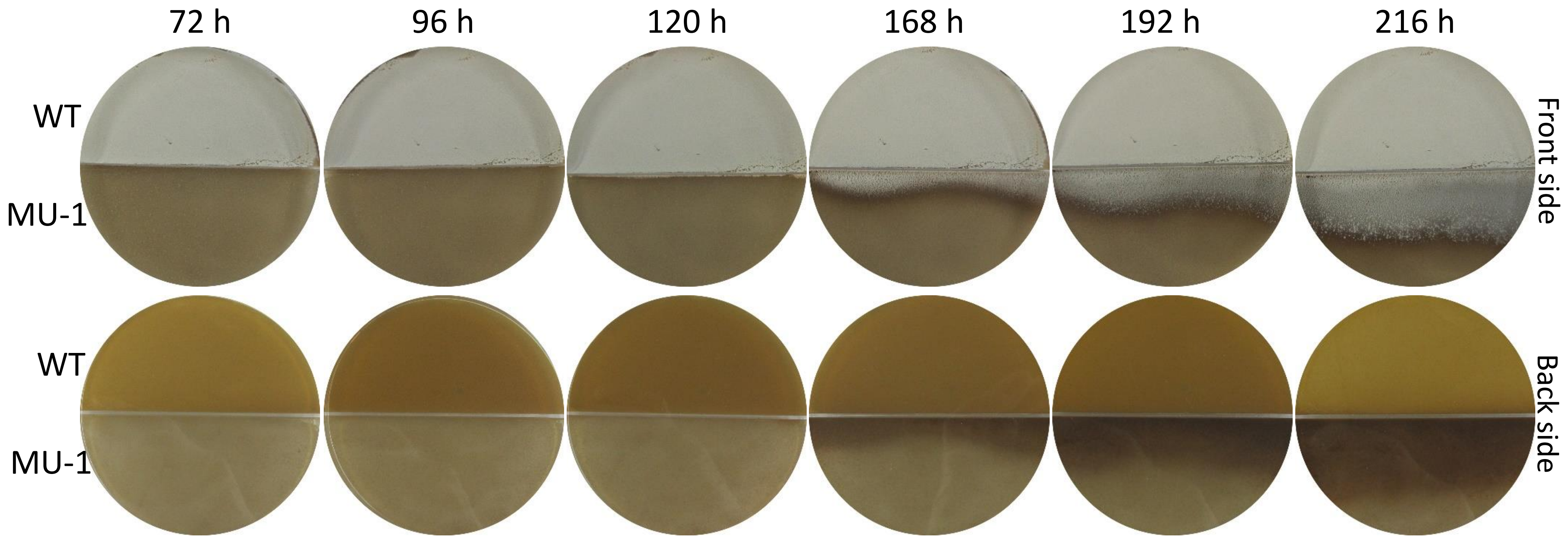

Fig. S6. Impact of the growth medium on the ability of the *S. venezuelae* wild-type strain ISP5230 to rescue MU-1**.** ISP5230 (WT) was grown on solid MS, and MU-1 was grown on solid N-Evans-CA medium in a petri dish with a physical barrier for the indicated times before photographing.

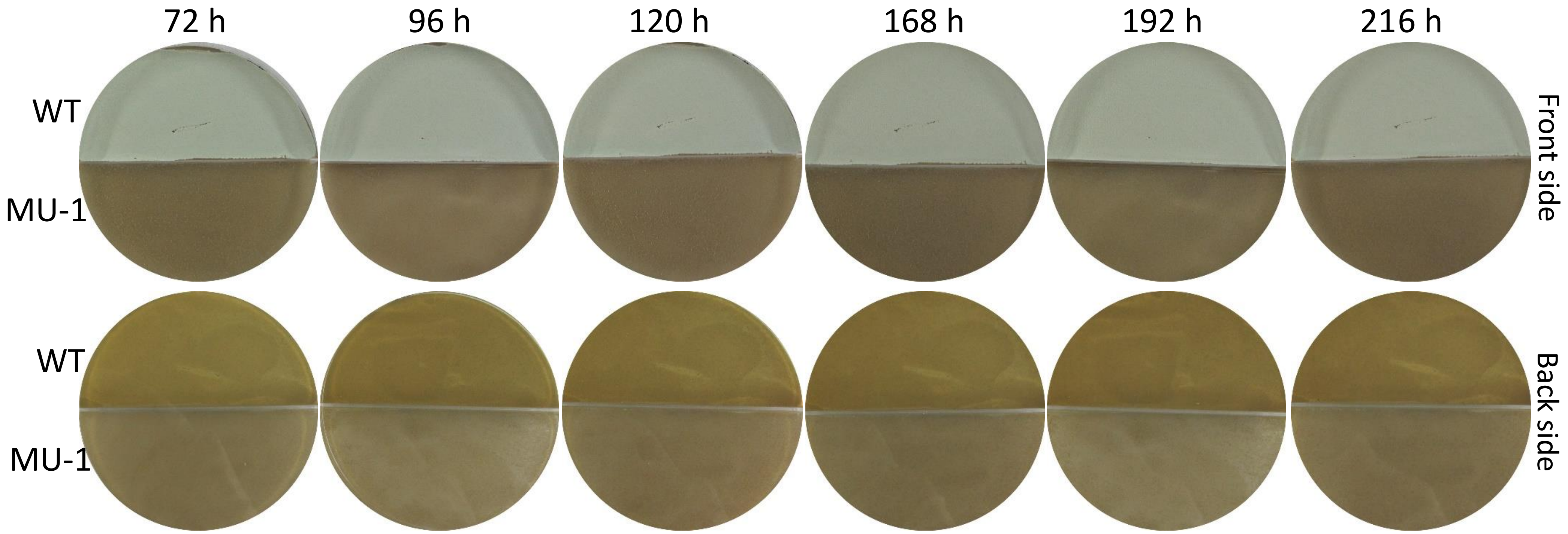

Fig. S7. Impact of the growth medium on the ability of the *S. venezuelae* wild-type strain ISP5230 to rescue MU-1**.** ISP5230 (WT) was grown on solid MYM, and MU-1 was grown on solid N-Evans-CA medium in a petri dish with a physical barrier for the indicated times before photographing.

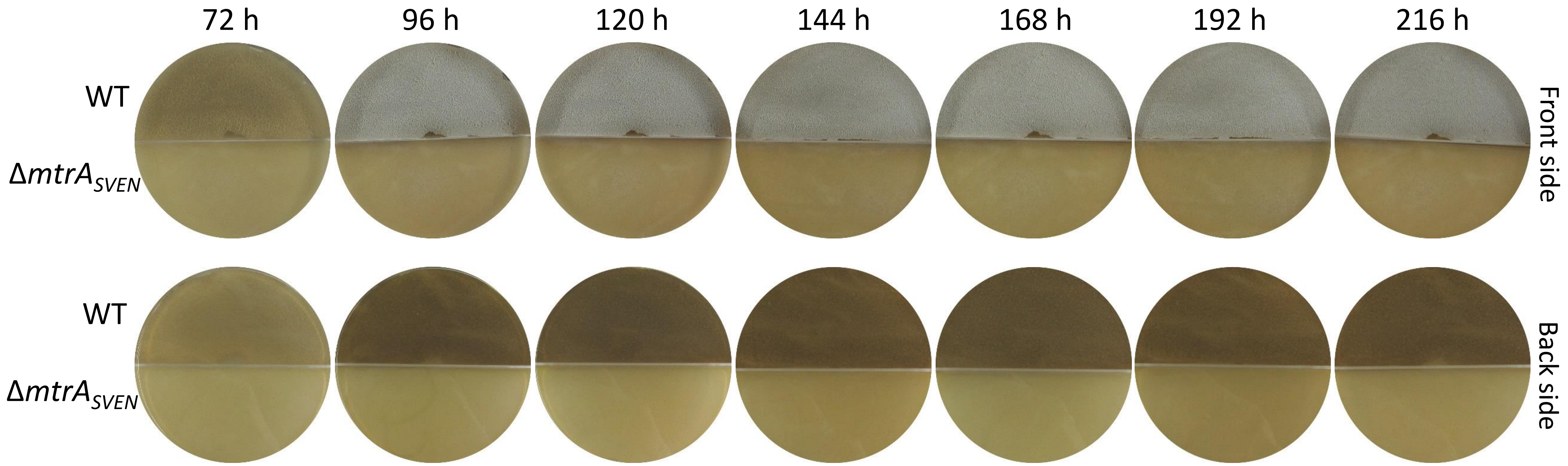

Fig. S8. Co-incubation analysis of the *S. venezuelae* wild-type strain ISP5230 and *S. venezuelae* mutant strain Δ*mtrA*$_{SVEN}$. ISP5230 (WT) was grown on solid N-Evans-CA medium, and Δ*mtrA*$_{SVEN}$ was grown on solid YBP medium in a petri dish with a physical barrier for the indicated times before photographing.

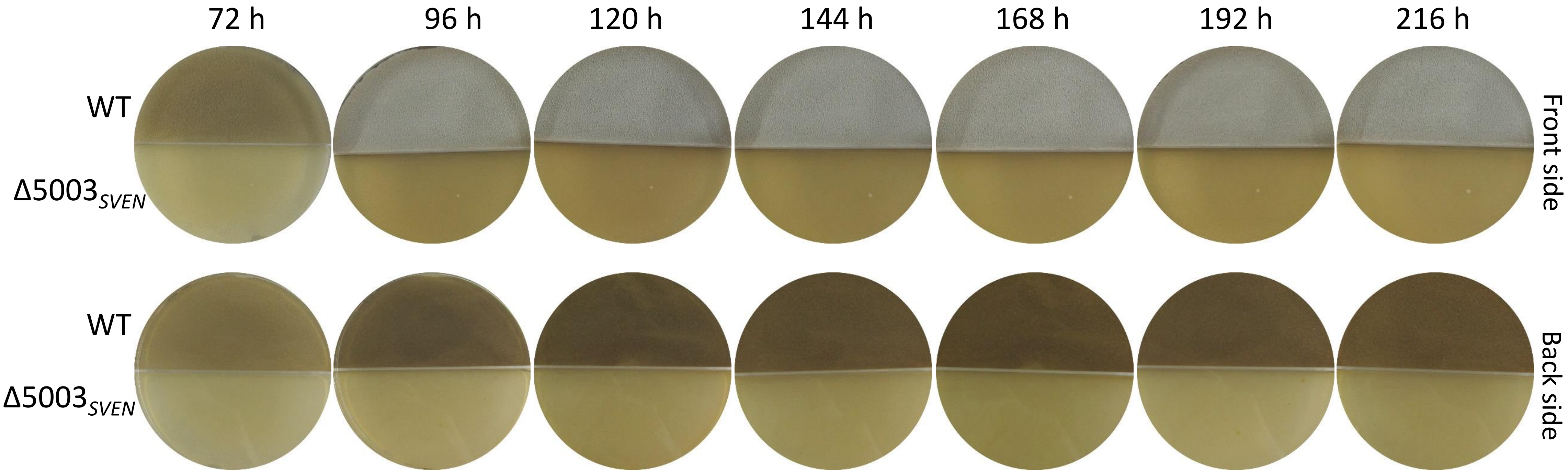

Fig. S9. Co-incubation analysis of the *S. venezuelae* wild-type strain ISP5230 and *S. venezuelae* mutant strain Δ5003$_{SVEN}$. ISP5230 (WT) was grown on solid N-Evans-CA medium, and Δ5003$_{SVEN}$ was grown on solid YBP medium in a petri dish with a physical barrier for the indicated times before photographing.

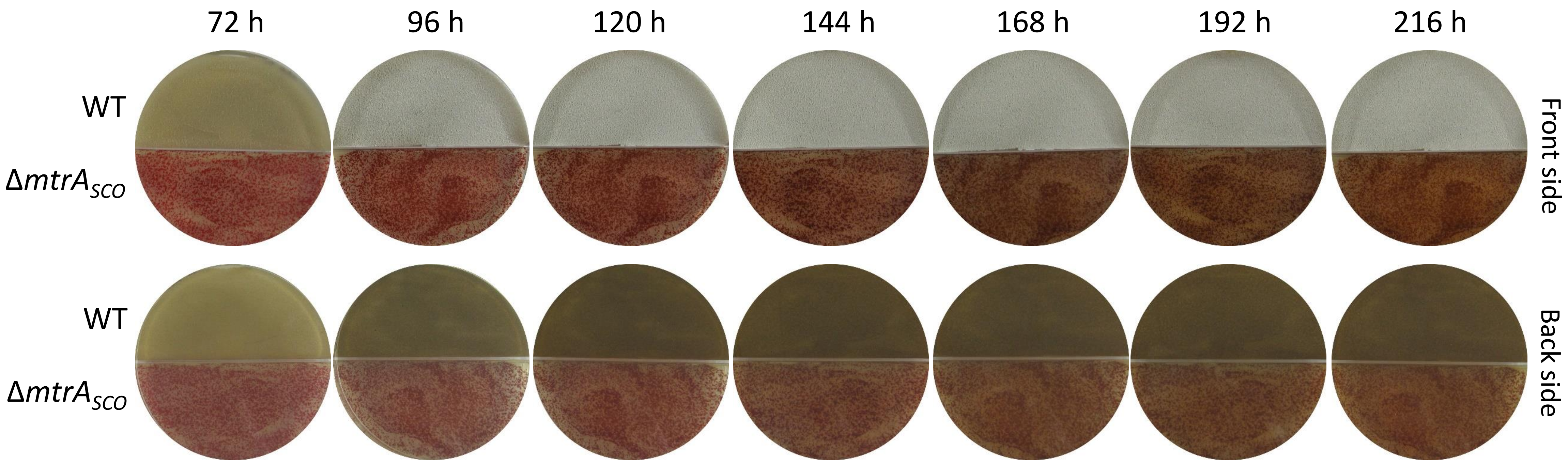

Fig. S10. Co-incubation analysis of the *S. venezuelae* wild-type strain ISP5230 and *S. colicolor* mutant strain Δ*mtrA*$_{SCO}$. ISP5230 (WT) was grown on solid N-Evans-CA medium, and Δ*mtrA*$_{SCO}$ was grown on solid YBP medium in a petri dish with a physical barrier for the indicated times before photographing.

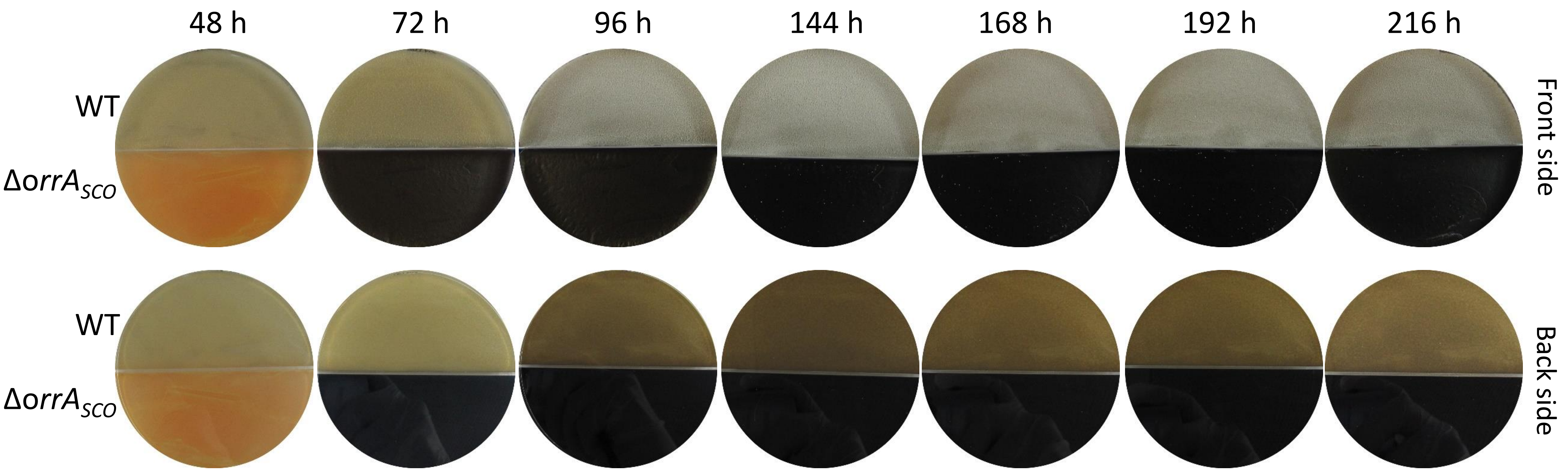

Fig. S11. Co-incubation analysis of the *S. venezuelae* wild-type strain ISP5230 and *S. colicolor* mutant strain Δ*orrA*$_{SCO}$. ISP5230 (WT) was grown on solid N-Evans-CA medium, and Δ*orrA*$_{SCO}$ was grown on solid YBP medium in a petri dish with a physical barrier for the indicated times before photographing.

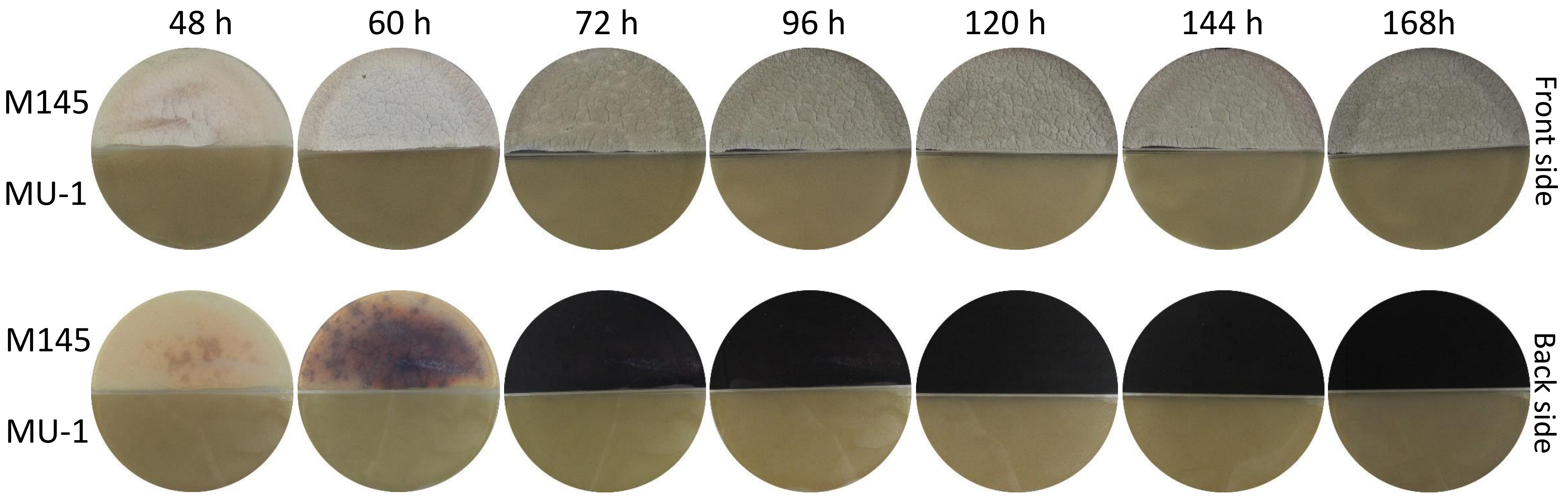

Fig. S12. Co-incubation analysis of *S. coelicolor* M145 and *S. venezuelae* strain MU-1. M145 and MU-1 were grown on solid N-Evans-CA medium in a petri dish with a physical barrier for the indicated times before photographing.

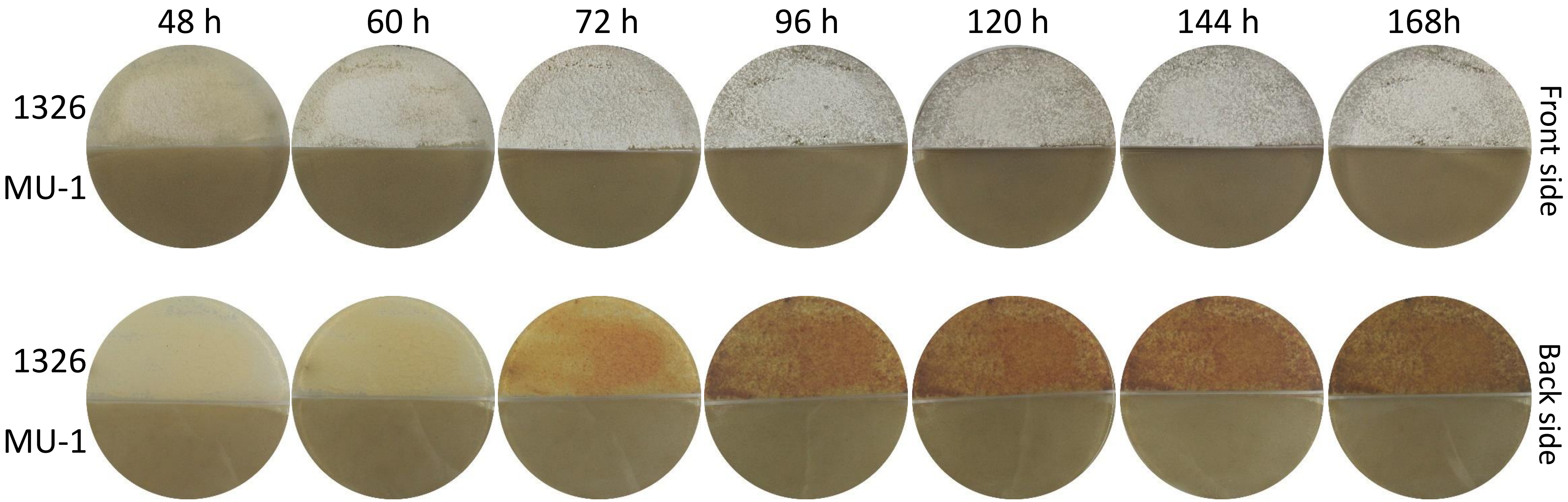

Fig. S13. Co-incubation analysis of *S. lividans* 1326 and *S. venezuelae* strain MU-1. 1326 and MU-1 were grown on solid N-Evans-CA medium in a petri dish with a physical barrier for the indicated times before photographing.

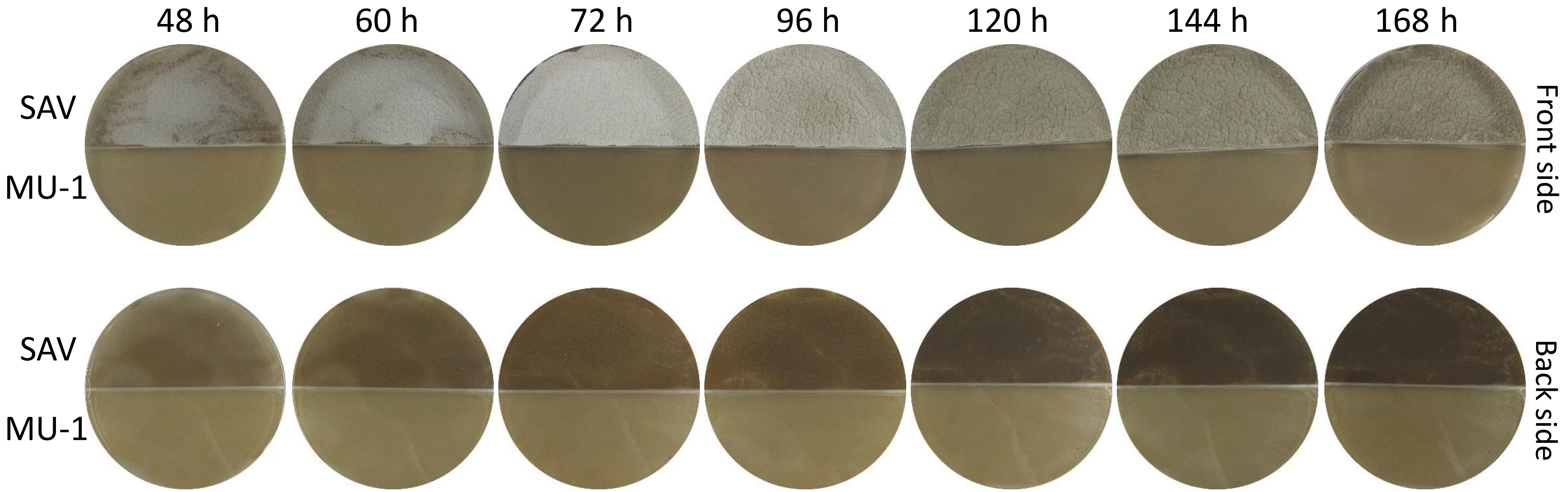

Fig. S14. Co-incubation analysis of *S. avermitilis* and *S. venezuelae* strain MU-1. *S. avermitilis* (SAV) and MU-1 were grown on solid N-Evans-CA medium in a petri dish with a physical barrier for the indicated times before photographing.

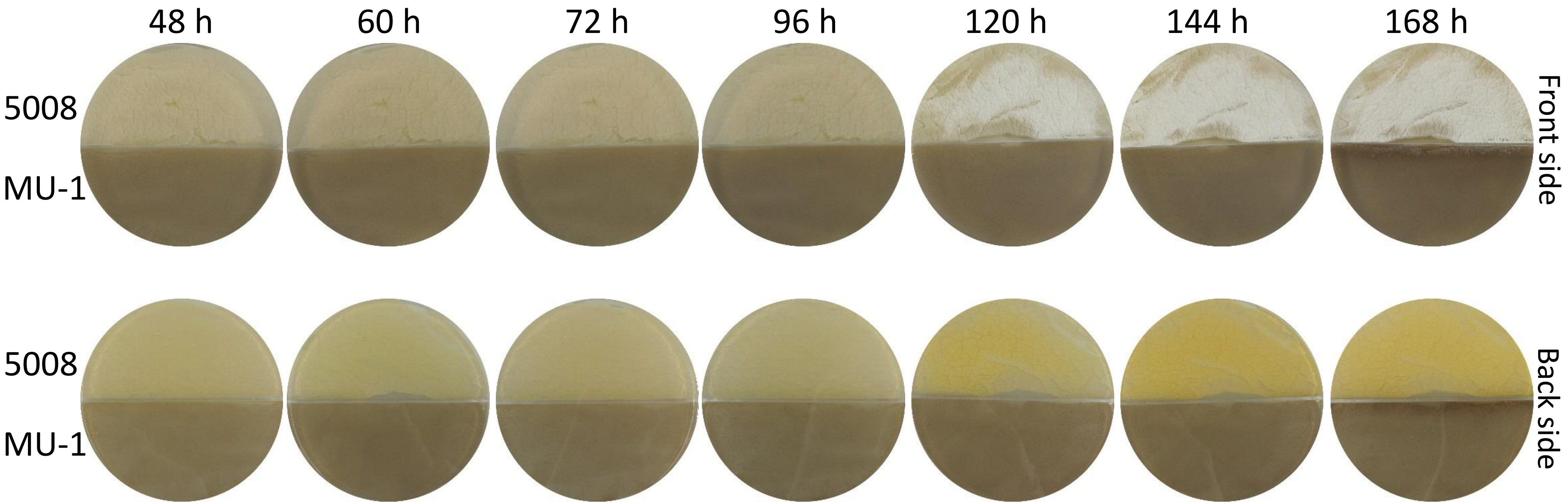

Fig. S15. Co-incubation analysis of *S. hygroscopicus* 5008 and *S. venezuelae* strain MU-1. 5008 and MU-1 were grown on solid N-Evans-CA medium in a petri dish with a physical barrier for the indicated times before photographing.

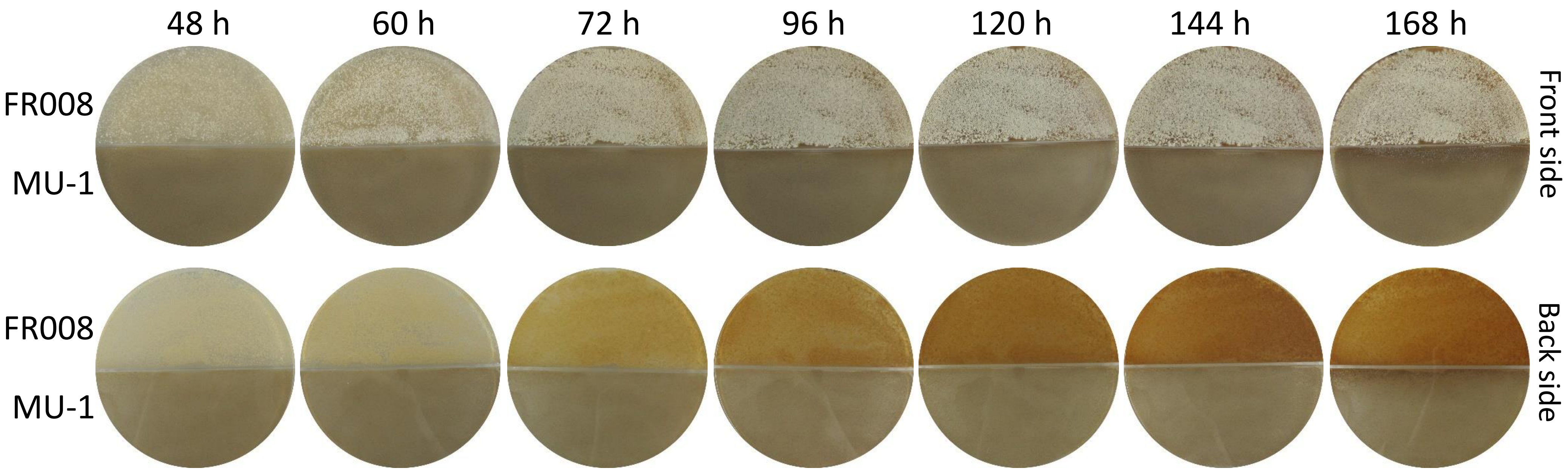

Fig. S16. Co-incubation analysis of *Streptomcyes sp.* FR008 and *S. venezuelae* strain MU-1. FR008 and MU-1 were grown on solid N-Evans-CA medium in a petri dish with a physical barrier for the indicated times before photographing.

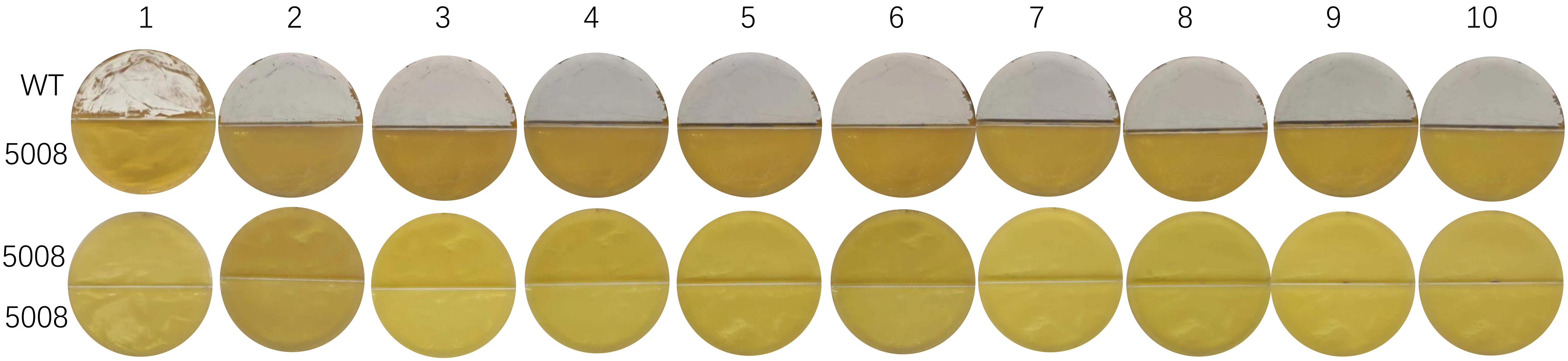

Fig. S17. Co-incubation analysis of the *S. venezuelae* wild-type strain ISP5230 and *S. hygroscopicus* 5008. ISP5230 (WT) was grown on solid MS medium, and *S. hygroscopicus* 5008 (5008) were grown on solid YBP in petri dishes with a physical barrier for the indicated times before photographing.

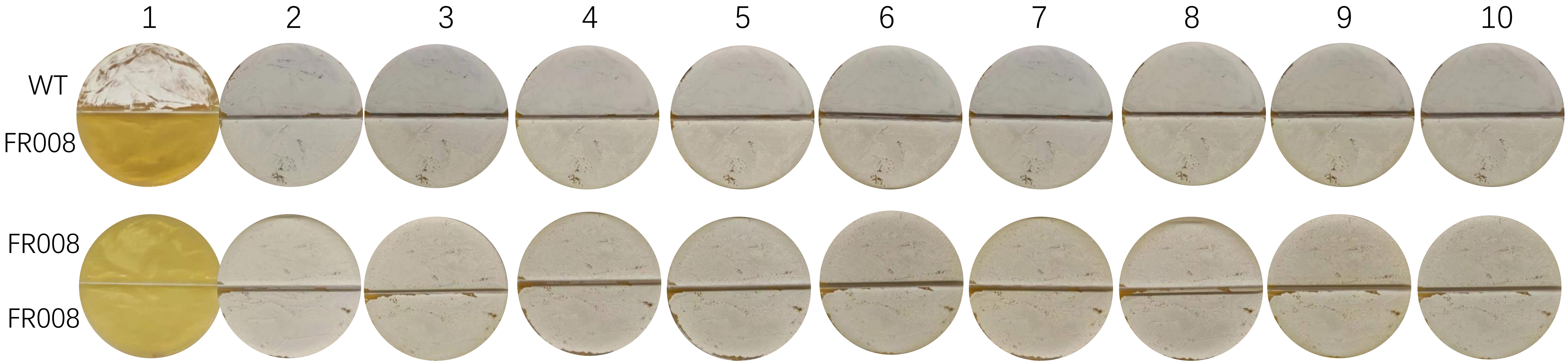

Fig. S18. Co-incubation analysis of the *S. venezuelae* wild-type strain ISP5230 and *Streptomyces sp.* FR008. ISP5230 (WT) was grown on solid MS medium, and *Streptomyces sp.* FR008 (FR008) were grown on solid YBP in petri dishes with a physical barrier for the indicated times before photographing.

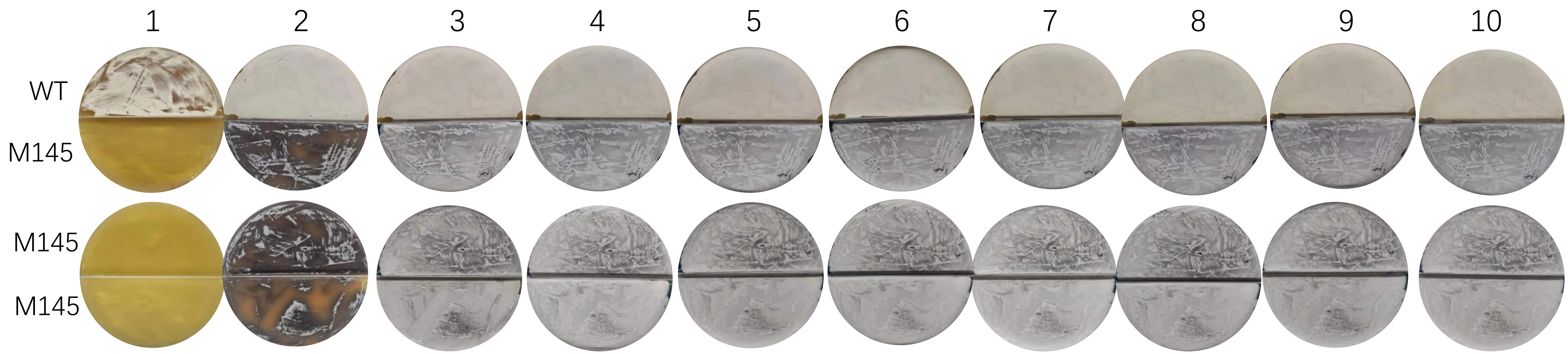

Fig. S19. Co-incubation analysis of the *S. venezuelae* wild-type strain ISP5230 and *S. coelicolor* M145. ISP5230 (WT) was grown on solid MS medium, and *S. coelicolor* M145 (M145) were grown on solid YBP in petri dishes with a physical barrier for the indicated times before photographing.

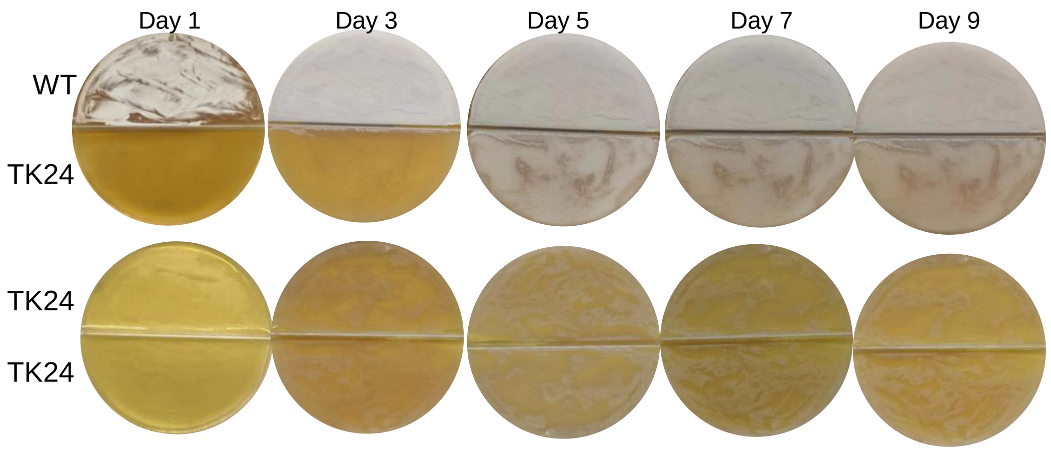

Fig. S20. Co-incubation analysis of the *S. venezuelae* wild-type strain ISP5230 and *S. lividans* TK24. ISP5230 (WT) was grown on solid MS medium, and *S. lividans* TK24 (TK24) were grown on solid YBP in petri dishes with a physical barrier for the indicated times before photographing.

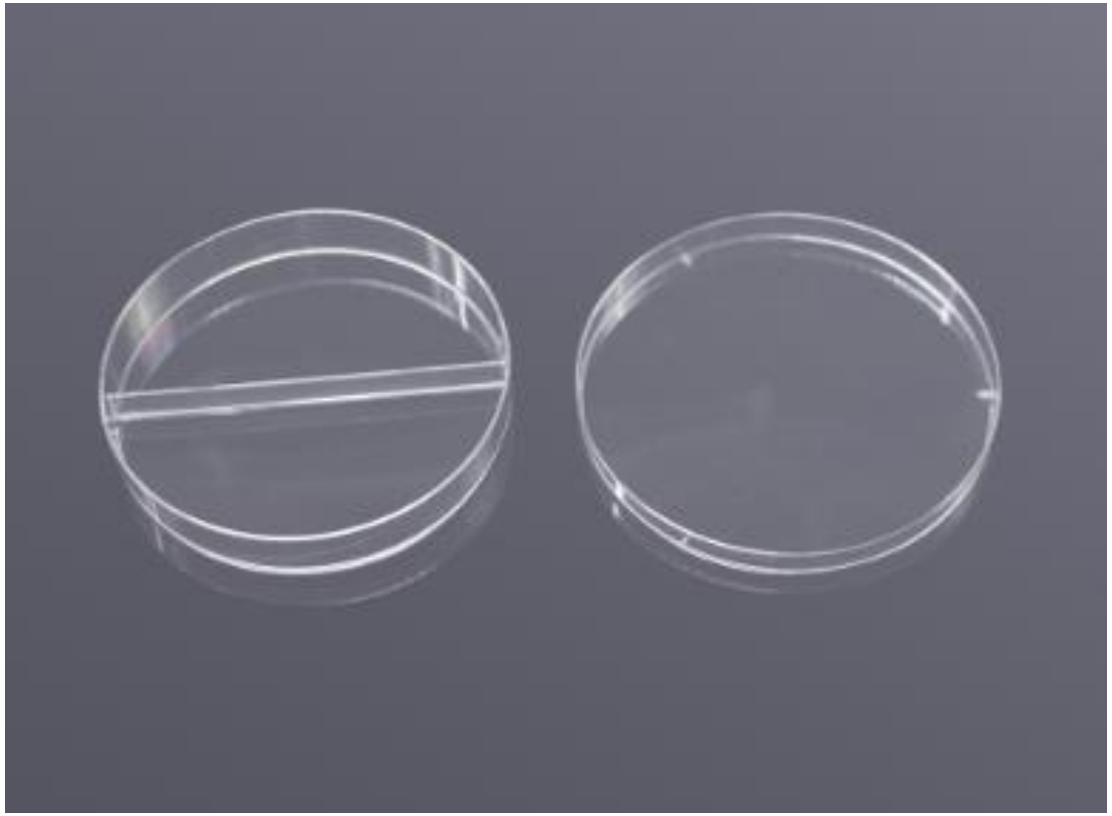

Fig. S21. Petri dish with physical barrier
